# Continuity of outcome assessment throughout the lifecycle of surgical research: mapping core outcome domains measured in early phase and late phase studies

**DOI:** 10.1186/s12893-025-03209-9

**Published:** 2025-10-10

**Authors:** Christin Hoffmann, Emma Sewart, Susanna Dodd, Sarah L. Gorst, Jane M. Blazeby, Kerry N. L. Avery, Shelley Potter, Rhiannon C. Macefield

**Affiliations:** 1 Bristol Medical School: Population Health Sciences, National Institute for Health and Care Research Bristol Biomedical Research Centre, Bristol Centre for Surgical Research, University of Bristol, Bristol, UK; 2 Bristol Medical School, Translational Health Sciences, University of Bristol, Bristol, UK; 3 Royal United Hospitals Bath NHS Foundation Trust, Bath, UK; 4 Department of Health Data Science, Institute of Population Health, University of Liverpool, Liverpool, UK; 5 University Hospitals Bristol and Weston NHS Foundation Trust, Bristol, UK; 6 North Bristol NHS Trust, Bristol, UK

**Keywords:** Outcomes, Surgery, Surgical innovation, Complex intervention evaluation, Core outcome set

## Abstract

**Background:**

Consistent outcome assessment in surgical research, from early phase studies (during introduction and refinement of new procedures) to late phase studies (to establish comparative effectiveness) needs improvement to ensure efficient and safe surgical care. This study explored the potential continuity of outcome domain assessment throughout the evaluation lifecycle of surgical interventions.

**Methods:**

Core outcome sets (COS) for late phase studies of surgical interventions were identified through COMET database searches. Core outcomes/outcome domains were extracted and mapped to core domains of a COS developed specifically for evaluating surgical innovation (COHESIVE COS). Outcomes/domains were categorised as “definite match” (clear similarity), “possible match” (potential similarity) and “no match” (no similarity) COHESIVE domain based on similarity in wording or meaning.

**Results:**

A total of 54 COS studies were included, yielding 573 core outcomes/domains. Most late phase core outcomes/domains (*N* = 519, 91%) showed clear or possible similarity. All late phase COS studies recommended measurement of COHESIVE domains ‘Intended benefits’ and ‘Expected and unexpected disadvantages’. Some late phase outcomes/domains also showed similarity with early phase COHESIVE domains, including ‘Problems with the device working’, ‘Patients’ experience’ and ‘Operators’/surgeons’ experience’. A minority of late phase outcomes/domains showed no similarity with COHESIVE domains (*n* = 54, 9%).

**Conclusion:**

High similarity between outcome domains recommended in early *and* late phase evaluations of surgical interventions demonstrates continuity of outcome domain assessment throughout the surgical innovation lifecycle is possible. Harmonising outcome measurement throughout the research pathway can streamline evaluation, enhancing access to beneficial treatment and improving early detection of harms.

**Supplementary Information:**

The online version contains supplementary material available at 10.1186/s12893-025-03209-9.

## Introduction

Surgery is vital for diagnosing, treating, and managing a wide range of medical issues [1]. The introduction of new surgical procedures and devices can occur outside of rigorous and formal research frameworks [2]. This is in contrast to new pharmaceuticals, which progress through an established pathway and are supported by staged early to late phase evaluation (see Table 1 for definitions). This implies early phase studies of surgical innovations are rare and often small, uncontrolled and exploratory in design [4]. New surgical interventions are commonly undergoing development and refinement following introduction which means early evaluation is essential to avoid innovations becoming widely adopted without robust comparative evidence from randomised controlled trials (RCTs) [4]. This is known to have severe impacts on patient safety [7–9]. There is an urgent need to improve the process by which new procedures/devices are widely adopted by improving robust evidence and thereby enabling the early detection of potential patient safety issues [10].

**Table 1:** Definitions

Surgical interventions
A surgical intervention is defined as an invasive procedure where purposeful/deliberate access to the body is gained via an incision, percutaneous puncture, where instrumentation is used in addition to the puncture needle, or instrumentation via a natural orifice. It begins when entry to the body is gained and ends when the instrument is removed, and/or the skin is closed. Invasive procedures are performed by trained healthcare professionals using instruments, which include, but are not limited to, endoscopes, catheters, scalpels, scissors, devices and tubes [3] There is no generally accepted definition of “early phase” or “late phase” studies in surgical research. The progression from early to late phase can be considered a continuum or as staged phases with no defined time frame. For the purposes of this study, we draw on multiple conceptualisations
Early phase studies
We adopted the conceptualisation of early phase consistent with stages 1 and 2 of the Idea, Development, Exploration, Assessment, Long-term follow-up (IDEAL) framework which can include first-in-human/case report (stage 1), prospective development (stage 2a) and exploration/feasibility studies (stage 2b) [4, 5]
Late phase studies
We adopted the Core Outcome Measures in Effectiveness Trials (COMET) Initiative’s conceptualisation of effectiveness trials which can include randomised or non-randomised clinical trials to evaluate safety and effectiveness of interventions, routine care and clinical audit studies We have adopted definitions proposed by Outcome Measures in Rheumatology (OMERACT) organisation to define outcome and outcome domain [6]
Outcome
The effect of treatment on a patient, which may be measured in a number of ways
Outcome domain Outcome domains represent the key areas of interest that are relevant to a particular research or clinical
field. For example, the outcome domain of physical function may include components such as the ability to perform activities of daily living, mobility, and range of motion. A domain is like a specific category or area of focus that researchers look at when they’re trying to understand a health condition
Potential continuity
Potential continuity refers to the semantic or conceptual alignment between outcomes/domains considered important to measure from early phase through to late phase studies. Please note, however, that true alignment or continuity (functional equivalence) cannot be established without the use of a validated framework which does not yet exist

Early detection and knowledge generation of benefit and harms necessitates consistent outcome measurement and reporting throughout the evaluation lifecycle, from early through to late phase evaluations. Unlike RCTs [11–13], processes for designing and reporting early phase studies are neither standardised nor comprehensive [14–21]. Early phase surgical research is characterised by a lack of well-established standards for measuring benefits and harms, potentially introducing heterogeneity and outcome reporting bias [22]. This means that different investigators may measure different outcomes or decide to report favourable outcomes or under-report important outcomes. Sub-optimal and selective outcome reporting in early and late phase evaluations of surgical procedures/devices may exaggerate benefits and underestimate harms [7, 23, 24]. In the United Kingdom, for example, novel transvaginal mesh implant surgery was widely adopted nationally due to early positive reports symptom relief, before high rates of harms and complications were reported later on [10].

Core outcome sets (COS) are a potential solution to reducing heterogeneity and outcome reporting bias [25, 26]. A COS is an agreed list of outcomes that should be measured and reported as a minimum in all clinical studies in specific areas of health or health care [26]. Traditionally, COS were intended to standardise outcome measurement for late phase RCTs that evaluate the comparative effects of interventions by measuring outcomes relevant to clinical effectiveness (e.g. benefits) and safety (e.g. harms) [26]. COS for surgical interventions have been developed for late phase evaluation of surgical interventions that are established, stabilised and no longer require modifications or refinement (i.e. Phase 3/4 trials). More recently, attention has turned to standardising outcome evaluation in early phase (i.e., Phase 1/2) studies of surgical innovations, where surgical interventions or devices are evolving, and their risks and consequences are unknown [16, 20, 27, 28]. In particular, recent work has identified outcome domains of importance to evaluating new surgical procedures and devices that are in their early phases of development [19, 21]. An international consensus study agreed on 8 broad outcome domains to be included in a COS for surgical innovation, recommended for use in all early phase studies of new procedures and devices (the core outcomes for early phase surgical innovation and devices/COHESIVE study) [23]. Robust methods for COS development [26, 29] were used to establish agreement on the COHESIVE COS, including systematic reviews, Delphi surveys and a consensus meeting [30]. This works seeks to improve the design and conduct of early phase surgical research [31].

Preventing avoidable patient harm from surgical innovations through early detection of their potential risks, disadvantages, and consequences requires continuous, comprehensive outcome evaluation throughout their lifecycle [10]. The relationship between core outcomes or core outcome domains measured in early and late phase evaluations of surgical procedures/devices has not yet been systematically explored. This study aimed to contribute to addressing this gap. Identifying which core domains are of importance throughout early *and* late phase evaluation of surgical interventions will help understand and improve continuity of outcome measurement and therefore improve evidence synthesis and efficient knowledge generation.

### Aims and objectives

The aim of this study was to explore how outcomes/domains identified in COS developed for late phase evaluations correspond with domains included in the COHESIVE COS for early phase studies, to assess the potential for continuity of outcomes throughout the evaluation lifecycle of surgical interventions.

A full description of the eight COHESIVE core domains is included in Additional file 1. Six core outcome domains included in the COHESIVE COS were considered of particular relevance to early phase evaluations (i.e., procedure success, modifications to the technique, operators’ physical, emotional and psychological experiences, unexpected advantages and disadvantages), whereas two core outcome domains were shared with effectiveness studies (i.e. expected advantages and disadvantages).

## Materials and methods

### Study design

A targeted review of a comprehensive COS database was undertaken to identify COS developed for late phase studies of invasive/surgical procedures. Core outcomes or core outcome domains (henceforth shortened to outcomes/domains) included within these were mapped to core outcome domains (henceforth shortened to domains) included in the COHESIVE COS developed for early phase surgical studies.

### Identification of COS studies

#### Eligibility criteria

Articles were included if they reported a COS developed for i) a specific type of invasive procedure and/or surgical device for any surgical specialty (with exception of orthodontics that do not involve instrumentation, e.g. application of braces to teeth); and ii) a disease or condition which includes surgical treatment as an option (e.g. COS for prostate cancer). No limitations were set in relation to the context in which the COS is intended to be used (e.g. for registries, in research or clinical practice) or the target population (e.g. patients of any age or sex).

Articles were excluded if they i) were published in non-English language; ii) did not report the full results of COS development (e.g. protocol papers); or iii) were not a COS development study (e.g., literature reviews, recommendations, definitions).

#### Search strategy

An electronic search of the Core Outcome Measures in Effectiveness Trials (COMET) Initiative database was performed (https://www.comet-initiative.org/Studies). This database was chosen because it is a comprehensive and up-to-date database of available COS populated by author registrations and annual systematic review updates to identify additional COS studies [32–35]. The integrated advanced search facility was used to identify all published COS developed for invasive procedures or surgical devices. The COMET Initiative online platform hosts resources for COS development and a database for ongoing and completed COS for comparative effectiveness trials.

An initial search was performed in February 2021 and updated in September 2022 to identify recent publications.

Existing search filters, pre-defined by the COMET database, were applied to identify COS development studies relevant to surgical interventions. The following available search filters were selected from standardised categories:

#### Nature/type of intervention

- Complex intervention
- Device
- Operative and non-operative management
- Postoperative management
- Procedure
- Surgery
- any, not specified (to capture studies potentially missed due to alternative indexing and ensure comprehensiveness of the search)

#### COS development study type

- COS for clinical trials or clinical research
- COS for practice
- COS for registry
- COS exclusively containing patient reported outcomes.

No filters were set for health area (disease category/name), target population (sex, age), method of COS development, interest holder involvement or publication year.

All search results were exported into Microsoft Excel and screened for eligibility against the inclusion and exclusion criteria in a two-step process outlines below.

#### Study selection

Two reviewer authors (ES, CH) independently assessed all titles and abstracts for eligibility. Any uncertainties with regards to eligibility were resolved in discussions with a third reviewer (RM). Full-text articles of potentially eligible studies were obtained from source journals. Eligibility was further assessed by one reviewer (ES or CH), with 10% of articles checked by a second reviewer (RM or SP). Reasons for exclusion were documented.

### Data extraction

A bespoke template for data extraction was created in Microsoft Excel (Additional file 2, Table S1).

Data extraction was performed by two reviewers (experienced health services researchers with expertise in systematic review methodology CH; and with a clinical background, ES). In addition, two reviewers with extensive experience in outcomes research (RM, an expert methodologist and SP, a breast cancer surgeon) independently performed double data extraction for 10% of included studies. Where insufficient detail was provided in the COS study, related publication(s) were accessed to complete data extraction (e.g. protocol papers).

### Mapping of outcomes/domains

Outcomes/domains extracted from the late phase COS studies were individually mapped to the eight broad domains included in the COHESIVE COS (see Additional file 2 for detail) to explore potential continuity. Existing methods for mapping COS outcomes/domains between routine care and research were used [36–38]. Specifically, mapping included examining similarities and discrepancies in the name and conceptual meaning of individual outcomes/domains and categorising these as “definite match” (as an indication of clear similarity or continuity), “possible match” (potential similarity) and “no match” (no similarity) (see Table 2 for detailed explanations). If the meaning of late phase outcomes/domains was unclear to the review team based on the verbatim description (i.e. the exact wording used in the source document), further contextual detail from the source publication was sought. Mapping of outcomes to broad COHESIVE domains was not mutually exclusive, meaning outcomes could be categorised as “definite” or “possible match” to more than one COHESIVE domain. For example, the verbatim outcome ‘Technical complications of the specific operation’ was categorised as a “definite match” to the COHESIVE domain ‘Expected and unexpected disadvantages’ as well as the domain ‘Problems with the device working’. Currently available methods allow for mapping to be based on semantic or conceptual similarity. They do not consider how outcomes/domains were operationalised, because associated core measurement sets are not consistently available. As a result, the findings should be interpreted as indicating potential continuity rather than providing evidence of confirmed functional equivalence.

**Table 2:** Categories of continuity based on matching/similarity between outcomes/domains extracted from late phase COS and early phase outcomes/domains

Definite match = outcomes/domains clearly represent the same/similar wording or meaning or referred to the same/similar concept. This implies a direct relationship between the late phase core outcome/domain and an early phase core domain(s). For example: ‘reduced pain’ was classified as a “definite match” to the ‘Intended benefits’ COHESIVE core domain
Possible match = outcomes/domains potentially represent the same/similar wording or meaning or referred to the same/similar concept, depending on the intended measurement of the outcome/domain not investigated in this study. This implies a likely relationship between the late phase core outcome/domain and an early phase core domain(s). For example: the domain ‘survival’ was classified as “possible match” because it could represent a benefit if survival was reduced, or it could represent a disadvantage if survival is shortened
No match = outcomes/domains did not represent the same/similar wording or meaning or referred to the same/similar concept. This implies there is no relationship between the late phase outcome/domain and any early phase domain

Mapping of outcomes followed a pre-written instruction manual (Additional file 3) to ensure consistency in the mapping process. The manual was based on existing guidance developed by co-authors SD and SLG that was used in previous core outcome mapping studies [36–38]. The existing guidance was adapted and refined to fit the context of this study through multiple rounds of calibration (i.e. the iterative process of refinement to ensure consistency and accuracy across reviewers) using a random pilot sample of 10% of included COS studies. During this process, four reviewers (RM, KA, SP, ES) independently and in duplicate mapped outcomes from the randomly selected COS studies. After each round, results were compared and discrepancy discussed collectively and the manual was refined accordingly. A fifth reviewer (CH) not involved in the manual refinement process applied the final manual to the same sample of included studies to test if outcome mapping results aligned with those from the other reviewers. This process ensured shared understanding and robustness of the mapping methodology. Outcome mapping for the remaining included COS studies was performed independently by two reviewers (ES, CH), with duplicate mapping performed for additional 5 included COS studies. When discrepancies occurred, additional reviewers (RM, KA) were consulted and where additional clinical input was required, a senior reviewer with relevant experience (SP) performed additional checks. Any discrepancies were resolved through discussion until a final decision was reached and agreed.

### Data analysis

Descriptive statistics were used to summarise the characteristics of the included studies, reported COS and their method of development, performed using statistical software (Stata version 17 and Microsoft Excel).

A quality assessment of included COS studies was not undertaken because there are currently no suitable tools available to evaluate the quality of COS development studies. In addition, the aim of this work was to synthesise outcomes/domains rather than critically appraise or evaluate the evidence for recommending COS.

## Results

### Included COS studies

**Fig. 1:**
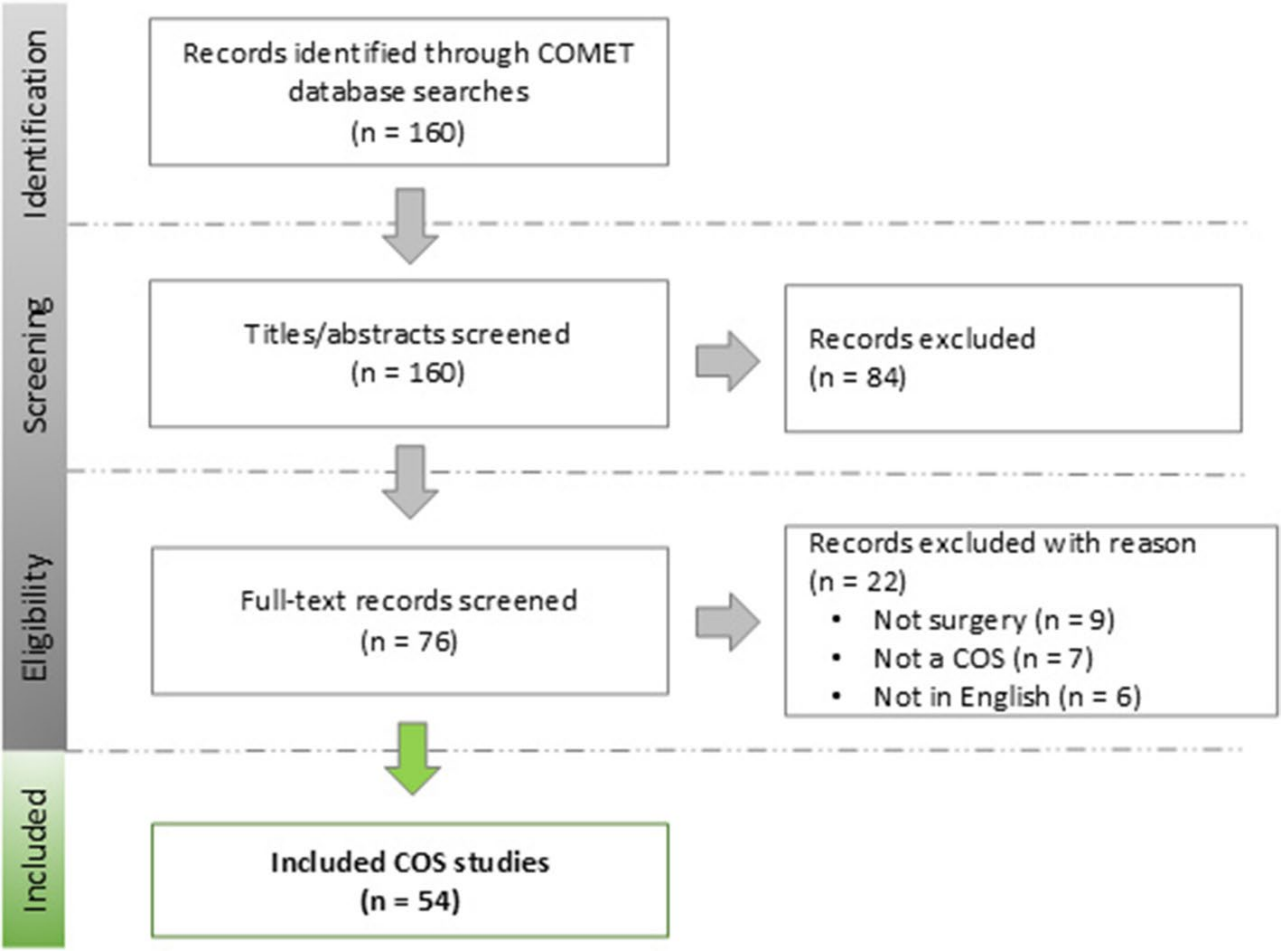
PRISMA flow diagram

A total of 54 COS studies were eligible for inclusion (see Fig. 1 for a PRISMA flow diagram). A detailed list of all included COS studies is available in Additional file 4.

The characteristics of included COS studies are summarised in Table 3. Out of 54 studies, most were published between 2017 and 2022 (*N* = 33, 61%). Neurology, orthopaedics, and trauma surgical sub-specialties were commonly represented (*N* = 17, 31%), followed by COS developed for cancer (*N* = 11, 20%). Obstetrics and gynaecology were least often represented in COS studies (*N* = 1, 2%). The majority of COS were relevant for invasive procedures not involving devices (*N* = 43, 79%), followed by COS for other types of invasive procedures, such as endoscopy, orthodontics, dialysis (*N* = 8, 15%). COS were most frequently intended to be used for clinical trials or research (*N* = 48, 89%) and a minority were developed for registries (*N* = 3, 5%). Detailed results for methods for item generation and consensus can be found in Additional file 2, Table S2.

**Table 3:** Characteristics of included COS studies (*N* = 54)

	*N *(%)
Year of publication	
2017–2022	33 (61)
2012–2016	16 (30)
2007–2011	3 (5)
< 2007	2 (4)
Disease category*	
Neurology, Orthopaedics, and Trauma	17 (31)
Cancer	11 (20)
Cardio-respiratory	9 (17)
Gastroenterology	7 (13)
Ears, Nose and Throat	2 (4)
Ophthalmology	2 (4)
Obstetrics and Gynaecology	1 (2)
Other	5 (9)
Target population**	
Adults	10 (18)
Children	8 (15)
Both	2 (4)
Not specified	34 (63)
Type of invasive procedure	
Surgical procedure only	43 (79)
Surgical procedure with device	3 (6)
Other invasive procedure	8 (15)
Setting for intended use***	
COS for clinical trials or clinical research	48 (89)
COS for practice and audit	13 (24)
COS for registry	3 (6)

^*^Disease categories initially based on COMET database and subsequently standardised and summarised

^**^Results are based on information specified in the source paper as recommended reporting standard by The COMET Handbook: version 1.0 [26]

^***^Results are based on information reported in the source paper. Classification adopted from COMET database; categories are not mutually exclusive

### Outcomes/domains included in late phase COS studies

A total of 573 outcomes/domains were extracted from included COS studies. The median number of outcomes/domains in each COS was 10 (range = 2–23) and most studies (*N* = 20, 37%) included between 6 and 10 outcomes/domains (see Table 4).

**Table 4:** Number of outcomes/domains per included COS study (*N* = 54)

Number of outcomes/domains per COS n	Number of studies N (%)
2–5	9 (17)
6–10	20 (37)
11–15	16 (30)
16–20	8 (15)
> 20	1 (2)

### Mapping of late phase outcomes/domains to COHESIVE COS

The results of mapping late phase outcomes/domains to early phase COHESIVE domains with illustrative examples are presented in Table 5. A list of further examples of extracted outcomes/domains is available in Additional file 2, Table S3.

**Table 5:** Mapping of late phase outcomes/domains to broad domains included in the COHESIVE COS

COHESIVE COS domain	Number of COS studies, *N* (%)*	Verbatim extracted outcomes/domains, *n* (%)**		
		categorised as a “definite match”	categorised as a “possible match”	Illustrative examples
Intended benefits	54 (100)	157 (27)	338 (59)	- sleep quality - the ability to eat and drink
Expected and unexpected disadvantages	54 (100)	299 (52)	180 (31)	- urinary incontinence - intraoperative complications - mortality
Overall desired effect achieved	45 (83)	21 (4)	92 (16)	- complete clearance of Actinic Keratosis - treatment success
Procedure completion success	12 (22)	3 (< 1)	16 (3)	- conversion to other procedure - inoperability
Problems with the device working	11 (20)	9 (2)	5 (< 1)	- device related complications - technical complications
Patients' experience	8 (15)	4 (< 1)	5 (< 1)	- patient satisfaction with treatment
Operators’/Surgeons' experience	2 (4)	0	2 (< 1)	- ease of intubation
Modifications to procedure, co-interventions or patient selection	0	0	0	n/a

^*^ % based on total number of included studies (*N* = 54)

^**^ % based on total number of extracted outcomes (*n* = 573)

#### Outcomes/domains with similarity across the evaluation lifecycle of surgical interventions

Most (*n* = 519, 91%) verbatim extracted late phase outcomes/domains were categorised as a “definite” or “possible match” to a domain included in the COHESIVE COS, indicating continuity between the late phase outcome/domain and an early phase domain(s).

Overall, the late phase outcomes/domains most commonly matched to the COHESIVE domains ‘Intended benefits’ and ‘Expected and unexpected disadvantages’ (*N* = 54, 100% of COS which included outcomes/domains matched to COHESIVE COS). Conversely, late phase outcomes/domains were most commonly categorised as “definite match” (intended benefits; *n* = 299, 52%) or “possible match” (expected and unexpected disadvantages; *n* = 338, 59%).

Extracted late phase outcomes/domains also frequently matched to the COHESIVE domain ‘Overall desired effect achieved’ (*N* = 45, 84%) but fewer outcomes were categorised as “definite match” (*n* = 21, 4%) or “possible match” (*n* = 92, 16%).

Late phase outcomes/domains were seldom matched to COHESIVE domains ‘Procedure completion success’ (*N* = 12, 22%) or ‘Problems with the device working’ (*N* = 11, 20%).

The least frequently matched late phase outcomes/domains related to COHESIVE domains ‘Patients’ experience with the innovative procedure’ (*N* = 8, 15%) and ‘Operators’/surgeons’ experience of performing the innovative procedure’ (*N* = 2, 4%).

#### Outcomes/domains that lack similarity across the evaluation lifecycle of surgical interventions

The minority (*N* = 54, 9%) of outcomes/domains extracted from late phase COS could not be matched to a COHESIVE COS domain for early phase studies. A full list of outcomes/domains identified as “no match” is included in Additional file 2, Table S4.

The majority of “no match” outcomes/domains were consistent with common data elements (e.g. micronutrient status, birthweight). These are routinely collected measures or descriptors to facilitate standardised reporting and evaluation in clinical trials or registries alongside, for example, patients’ socio-demographic information or physical measures [39, 40].

“No match” outcomes/domains also commonly related to measures of resource use (e.g. days spent admitted to hospital/intensive care per year, hospital days). These are measures relevant to health system efficiency or economic metrics often used for cost-effectiveness analyses in trials [41].

No late phase outcome/domain were matched to the COHESIVE COS domain ‘Modifications to procedure, co-intervention or patient selection’.

## Discussion

To our knowledge, this study is the first to explore the potential continuity of outcome domain assessment throughout the evaluation lifecycle of surgical interventions to improve evaluation of surgical procedures. A COMET database search was performed to identify 54 COS studies relevant to invasive surgical procedures/devices for intended use in late phase studies (including trials, routine care and audit). A total of 573 core outcomes/domains were extracted and mapped to the COHESIVE COS, an eight-item COS specifically developed to evaluate early phase surgical interventions. Results showed high similarity/continuity between late phase COS and the COHESIVE COS for most (91%) extracted outcomes/domains. This highlights outcome domains of importance during the evaluation of invasive interventions at all stages of the research pathway and the potential for early phase outcome domains to inform late phase outcome domain measurement and reporting. Some 54 (9%) outcomes/domains that were not matched to any early phase domain and frequently represented data items routinely collected in late phase studies (e.g. common data elements and resource use metrics). Overall, it is recommended that all studies assessing surgical interventions should measure and report outcomes relevant to early *and* late phase evaluation. The findings are useful to optimise continuity in the measurement of outcomes/domains throughout the evaluation lifecycle of surgical interventions and thereby promote the comprehensive and efficient generation of evidence about promising and harmful innovations.

### Results in the context of literature

The finding that late phase outcomes/domains were most commonly matched to ‘expected and unexpected disadvantages’ is not surprising, as these types of outcomes are important in late phase evaluations to establish whether the surgical intervention is safe. Extracted outcomes/domains commonly referred to known adverse events or disadvantages and no outcome/domain specifically related to *unknown* harms or disadvantages. This suggests that disadvantages reported in late phase COS are largely expected and may not include *unexpected* disadvantages which generally occur less commonly [16, 19–21]. Recent research found that harm and adverse event outcomes are highly prevalent in COS development and are of great importance [42]. Given that uncertainty is inherent to early phase surgical research, it is essential to measure the unintended consequences, risks and harms of surgical interventions [43]. Thereby, the selection of relevant harm outcomes should consider relevant methodological approaches (e.g. OMERACT filter) or outcome type (pooled vs. individual) [42, 44]. Continuous measurement throughout the evaluation lifecycle would be beneficial to monitor trends of unexpected disadvantages which can ultimately inform conclusions about intervention stability.

Results of outcome mapping revealed some discordant outcomes/domains. As anticipated, the COHESIVE domain ‘Modifications to procedure, co-interventions or patient selection’ was not represented in late phase outcomes/domains. This is likely due to the expectation that invasive interventions have stabilised by the point of definitive late phase evaluation and usually no further procedure refinement is taking place [22]. It is important to note, however, that modifications may still hold relevance in late phase evaluations because strict standardisation in studies involving surgical intervention can be neither feasible nor desirable [4, 45]. In general, there are other complexities in outcome methodology arising from multi-component, complex interventions. Contextual factors such as variations related to surgical specialty, patient anatomy, hospital processes or surgeon expertise/preference lead to inevitable variations across studies and even within interventions considered well-established. These factors require careful monitoring to understand how they may influence the evaluation of surgical interventions [46]. Ensuring intervention fidelity and defining/monitoring how the delivery of surgical interventions is standardised is critical for accurate and robust evidence generation [47, 48]. Advances in the development of quality assurance methods have been made [47] and the continuous assessment of modifications may give important details about the level of intervention fidelity/stability throughout the evaluation lifecycle. This signifies the importance of transparent and consistent data capture to monitor contextual factors, and underscores the need for further methodological advancements in this area.

Similarly, extracted outcomes/domains not represented in any of the early phase domains (i.e. those identified as “no match”, *N* = 54, 9%) were also anticipated. The majority of identified outcomes/domains were aligned with routinely reported descriptors (common data elements) and health economic measures (resource use), which are not considered in the COHESIVE COS. This is because many COS developers consider common data elements relevant data items to report in studies, but do not consider these an outcome [49]. Resource use was not considered at the time of the COHESIVE COS development because health economics methods for early phase studies is currently not well understood [50]. The development of methods for measuring and reporting resource use in early phase studies, however, is the subject of future planned work within the National Institute for Health and Care Research Biomedical Research Centre.

COHESIVE domains relevant to patients’ and surgeons’ experience of surgical innovation were rarely represented in late phase COS. This COHESIVE domain is considered innovation-specific and it is therefore not surprising that late phase outcomes/domains did not cover self-reported perceptions or experiences related to an innovative surgical procedure or device. Whilst patient and clinician-reported outcome measures are undoubtedly recognised as vital to assessing effectiveness of interventions, experience measures are often not considered in COS development [51]. Recent evidence suggests there is a demand and need for the inclusion of patient-reported experience measures in surgery to optimise patient-centred care [52, 53]. Likewise, physical hardship (e.g. musculoskeletal pain) and psychological stress factors (e.g. distractions in the operating room) have been reported to influence the surgeon’s performance and patient safety [54, 55]. Ultimately, considering both patient and surgeon experience measures in future COS development may enhance evaluation of invasive interventions.

### Implications

There is growing interest in outcome standardisation across many interventions and disease areas [56]. Funders, trialists and journals endorse the use of COS [57], which led to an increase COS development across research, quality improvement and performance assessment [25, 26, 33]. Findings in this study reflect this trend, with the majority (61%) of included COS studies published between 2017 and 2022. The uptake of COS in clinical practice and research, however, remains low [58, 59]. Possible reasons might include lack of their perceived awareness and relevance due to not involving all interest holders during COS development, as well as a lack of concordance with guidelines and detailed implementation guidance, hindering their practical application [59, 60]. To enhance impact and utility of COS, developers should consider the full evaluation lifecycle of invasive interventions and all their potential interest holders from the outset.

The current study demonstrates the feasibility of aligning outcomes across phases of surgical intervention evaluation, suggesting the need to integrate insights from both early and late phases. This complements recommendations to not restrict the use of COS to trials and reviews, instead COS “*should be considered across the research evidence creation*” [61]. The Medical Research Council’s guidance for the evaluation of complex interventions highlights their cyclical nature and the importance of considering the full intervention lifecycle [62]. Inclusion of outcomes/domains relevant to both early *and* late phase evaluations of surgical interventions is therefore recommended in future COS development to better support coherent and consistent evidence generation and synthesis.

One practical way to include outcomes/domains specific to early and late phase studies would be to adopt modular approaches to COS development. Such approaches have previously been used to widen their scope and may provide a solution for incorporating early and late phase outcomes/domains. For example, some COS studies separated outcomes by time (e.g. short vs. medium term studies [63]), or stratified relevance of outcomes by intervention context (e.g. all treatments vs. surgery only [64]). Likewise, COS including different tiers have been suggested to incorporate outcomes of varying levels of perceived interest holder importance [65]. Such approaches could be considered to include separate sets of outcomes particularly relevant to early and late phase studies.

This study has potential relevance for existing health outcomes databases used to evaluate invasive interventions (e.g. The National Joint Registry in Orthopaedics [66], or The United Kingdom National Flap Registry for plastic surgery [67]). The need for standardised, continuous and long-term outcome assessment throughout the lifecycle of invasive interventions has been highlighted [68, 69] and formed a key recommendation of the 2021 Independent Medicines and Medical Devices Safety Review, chaired by Baroness Cumberlege (Recommendation 8: The creation of a central patient-identifiable database) [10]. It is suggested that investigators consider implementing routine assessment of outcomes/domains that may be of relevance to the full evaluation lifecycle (‘Intended benefits’, ‘Expected and Unexpected disadvantages’, ‘Overall desired effect achieved’), agreed through appropriate multi-disciplinary consensus in context. This may facilitate early detection of benefits and harms, ultimately improving patient safety and optimising evidence-based surgical care.

The COHESIVE COS was developed to support standardised outcome measurement across the evaluation lifecycle. It includes broad domains relevant to a range of surgical interventions which correspond with late phase outcomes/domains that vary in specificity and target context. While considering outcome selection at this broader (domain) level enables flexibility throughout the evaluation lifecycle, it is important for investigators to consider relevant context-specific outcomes and how these outcomes may evolve as evaluation progresses. In particular, investigators and relevant interest holders should carefully review outcomes/domains included in early and late phase COS to ensure they have fully considered the breadth of outcomes relevant to measure and propose additional relevant outcomes (e.g. those identified in this study as ‘no match’).

### Limitations

This study used robust methods to map early and late phase outcomes/domains extracted from a comprehensive list of COS development studies relevant to surgery. Limitations of this review should be noted. The systematic database searches were undertaken using the COMET database as the only source. Identification of COS studies relied, therefore, on studies being indexed in this database and it is acknowledged that any COS development studies that were not recorded in the COMET database (including very recent studies or COS for highly specialised surgical procedures) may therefore have been missed. Whilst this is a limitation of our search strategy, the COMET database entries are populated using a well-established, robust, annual systematic review of published COS identified from searching Medline and Scopus databases [25, 32–35, 70]. As such, this provides a comprehensive and up-to-date database of available COS. Whilst searching additional databases may have potentially identified a small number of additional COS studies, we do not feel that this would change the main conclusions of our study. The aim of this study was to explore how early and late phase COS domains may correspond and detect potential for continuity across outcome assessment.

The generalisability of findings of this study are limited due to the uneven representation of surgical specialties. The majority of outcomes/domain were extracted from COS studies developed for conditions related to ‘Neurology, Orthopaedics, and Trauma’ (*N* = 17, 32%) or ‘Cancer’ (*N* = 11, 20%). We did not perform further analyses to explore the implications of this overrepresentation may affect potential continuity. Generalisability is further limited due to the inclusion of COS studies only published in English language. This decision was due to limited resources for translating non-English articles. This resulted in the exclusion of six studies, representing an only small proportion of identified COS studies. This language restriction, however, may have introduced bias by underrepresenting COS study developed in non-English speaking regions or contexts.

We acknowledge an additional limitation related to the lack of quality appraisal. No formal quality appraisal was undertaken because there is currently no validated or widely accepted tool for appraising the quality of COS development studies. In the absence of an established criteria for “low quality” in this context, we felt that applying subjective criteria risked introducing additional bias and favoured maximising the breadth of identified COS and their outcomes/domains in the literature. Moreover, this study did not intend to provide recommendations or scrutinise the development of existing COS for evaluations of surgical procedures/devices. Instead, the focus of this work was to examine potential continuity of late phase and COHESIVE COS outcomes/domains. We therefore did not consider the level of COS study quality informative to answering the research question. We recognise that a range of higher and lower quality COS studies may have been included in our study, meaning mapping results include outcomes/domains from COS developed with less rigour. By including studies of low methodological quality, the included COS could also be low quality, meaning they could not include core outcomes/domains most important to relevant interest holders. The impact of this on conclusions about potential continuity is not known, but could suggest an decrease or increase of potential continuity. However, we expect the potential impact to be low because, for the purposes of the mapping exercise, it was important to capture the a wide spectrum of outcomes/domains proposed in the literature, regardless of how robust the underlying consensus processes were.

Limitations in relation to the process of mapping outcomes are recognised which need to be considered when interpreting the findings. There are no established or agreed methods for examining continuity of outcomes/domains between early and late phase studies. Existing methods for mapping outcomes/domains in routine care and research [36–38] were considered most suitable to answer the research question. These were adapted in collaboration with the authors of the existing methods (SD, SLG) and may inform future work to establish methods for mapping outcomes. Despite efforts to maximise the reliability of the mapping process (e.g., use of multiple highly experienced reviewers representing the perspectives of methodologists, clinicians and health service researchers, and use of a mapping manual), it is acknowledged that the process of mapping outcomes involves a natural degree of subjectivity. We were unable to provide quantitative detail about any disagreements during the double coding process as these were not recorded. Similarly, we did not calculate inter-rater reliability statistics due to insufficient number of double coded outcomes/domains, which means the reliability of the mapping process is uncertain. Due to In addition, the aim of this study was to identify all plausible matches and did not consider them mutually exclusive which may overestimate the degree of matches within a COHESIVE domain. Further, the approaches used to map outcomes/domains do not take into consideration the timing of measurement and does not distinguish between short and long-term outcomes. We have not undertaken a substantive analysis about how different measurement instruments and timing of measurement may affect conclusions about potential continuity. The current exploration of potential continuity between early and late phase studies may therefore not accurately reflect the exact degree of continuity between outcomes/domains (i.e. functional equivalence) and does not take into account the effects of heterogeneous outcome measurement. Establishing robust methods for the process of mapping outcomes/domains alongside approaches for analysing effects of measurement variability would be important for the COS community and may be explored in future research. This work should include the development of a framework to evaluate the functional equivalence of outcomes/domains and should be fully co-developed and validated with all relevant interest holders (e.g., patients/carers, surgeons, academics, allied health professionals, representatives from regulatory bodies, funders and journals). Future methods should also track the agreement between raters during the mapping process to be able to calculate inter-rater reliability statistics.

Furthermore, the process of mapping considered semantic and conceptual similarity of outcomes/domains but did not account for the variability in how they are operationalised. This was because studies reporting the content of a COS frequently only describe the recommended outcomes/domains, without providing detail on how they should be measured in the context of a study or clinical practice [71]. Dedicated methods for the development of core measurement sets are well-recognised within the COS community [72]. Core measurement studies are often conducted after COS development as standalone project, rather than alongside, resulting in a gap between outcome definition and their consistent implementation in research and practice. Mapping therefore occasionally entailed challenges in deciding the level of relationship between outcomes/domains. For example, categorising the outcomes/domains as either “definite match” or “possible match” was challenging in stances where studies reported key outcomes of specific procedures without additional contextual knowledge. Specifically, for the outcome “range of movement of hip flexion” in a COS for total hip arthroplasty, it was difficult to determine whether this relates to a “possible match” or “definite match” to the COHESIVE domain ‘overall desired effect achieved’ because of the lack of procedure-specific information within the COS development study. To address this, the study involved constant team discussions between clinical and non-clinical researchers with varying expertise which ensured that decisions resulted in consensus agreement between the multi-disciplinary team members. Nevertheless, the general limitation of variability in COS applications means that our conclusions only reflect continuity based on semantic/conceptual alignment of outcomes/domains, rather than equivalence of their measurement or operationalisation in different contexts. Exploring the consistency of how outcomes/domains are measured across studies, including their alignment in early and late phase studies, is an important avenue for future research.

### Conclusion

This study explored continuity of outcomes/domains during early and late phase evaluation of surgical interventions and suggests domains of importance throughout the evaluation lifecycle. Improved continuity of outcome assessment is needed to facilitate efficient evaluation of surgical interventions to ultimately achieve safe and effective patient care. COS developers should consider adopting new best practice that explicitly promote the alignment of outcomes/domains across late and early phase evaluations.

## Supplementary Information

Supplementary material 1.

## Data Availability

All data generated or analysed during this study are included in this published article and its supplementary information files.

## Abbreviations

COHESIVE: Core Outcomes for Early Phase Surgical Innovation and Devices
COMET: Core Outcome Measures in Effectiveness Trials
COS: Core Outcome Set
IDEAL: Idea, Development, Exploration, Assessment, Long-term framework
OMERACT: Outcome Measures in Rheumatoid Arthritis Clinical Trials
PRISMA: Preferred Reporting Items for Systematic Reviews and Meta-Analyses
RCT: Randomised controlled trial
