# Supplementary material for: Continuity of outcome assessment throughout the lifecycle of surgical research: mapping core outcome domains measured in early phase and late phase studies
Source: BMC Surg. 2025 Oct 10;25:473. doi: 10.1186/s12893-025-03209-9 (PMC12512511; doi:10.1186/s12893-025-03209-9)
Supplement: Supplementary file 1 — Supplementary material 1. [file 12893_2025_3209_MOESM1_ESM.zip › Additional file 4 - Included COS studies.pdf]

Additional file 4

Table S2: List of all included COS studies

**Authors**	**Year**	**Title**	**Disease category**	Setting for intended use
Hinkelbein, J. et al	2019	Outcomes in video laryngoscopy studies from 2007 to 2017: systematic review and analysis of primary and secondary endpoints for a core set of outcomes in video laryngoscopy research	Cardio-respiratory	
Manera, K. E. et al	2020	Establishing a Core Outcome Set for Peritoneal Dialysis: Report of the SONG-PD (Standardized Outcomes in Nephrology–Peritoneal Dialysis) Consensus Workshop	Other	
Leo, D. G. et al	2020	The outcomes of Perthes' disease:<br>Development of a core outcomes set for clinical trials in Perthes' disease	Neurology, Orthopaedics & Trauma	
Sherratt, F. C. et al	2020	Core outcome set for uncomplicated acute appendicitis in children and young people	Gastroenterology	
Ambler, G. K. et al	2020	Development of Core Outcome Sets for People Undergoing Major Lower Limb Amputation for Complications of Peripheral Vascular Disease	Cardio-respiratory	
Srikandarajah, N. et al	2020	Cauda Equina Syndrome Core Outcome Set (CESCOS): An international patient and healthcare professional consensus for research studies	Neurology, Orthopaedics & Trauma	
Aliki Tsichlaki<br>Kevin O'Brien<br>Philip E. Benson<br>Zoe Marshman<br>Ama Johal<br>Fiorella B. Colonio-Salazar<br>Nicola L. Harman<br>Padhraig S. Fleming	2020	Development of a core outcome set for use in routine orthodontic clinical trials	Other	
Fleur A. ten Tije, Robert J. Pauw, Joze C. Braspenning, Raphael J. B. Hemler, Annette J. ter Schiphorst, Erik F. Hensen, Lisa van der	2020	Uniform Registration Agreements on Cholesteatoma Care: A Nationwide Consensus Procedure	ENT	

Putten, Sophia E. Kramer, and Paul Merkus				
Hodgson CL1, Burrell AJC1,2, Engeler DM1, Pellegrino VA2, Brodie D3, Fan E4,5; International ECMO Network.	2019	Core Outcome Measures for Research in Critically Ill Patients Receiving Extracorporeal Membrane Oxygenation for Acute Respiratory or Cardiac Failure: An International, Multidisciplinary, Modified Delphi Consensus Study	Cardio-respiratory	
Balakrishnan, K. Sidell, D. R. Bauman, N. M. Bellia-Munzon, G. F. Boesch, R. P. Bromwich, M. Cofer, S. A. Daines, C. de Alarcon, A. Garabedian, N. Hart, C. K. Ida, J. B. Leboulanger, N. Manning, P. B. Mehta, D. K. Monnier, P. Myer, C. M., 3rd Prager, J. D. Preciado, D. Propst, E. J. Rahbar, R. Russell, J. Rutter, M. J. Thierry, B. Thompson, D. M. Torre, M. Varela, P. Vijayasekaran, S. White, D. R. Wineland, A. M. Wood, R. E. Wootten, C. T. Zur, K. Cotton, R. T.	2019	Outcome measures for pediatric laryngotracheal reconstruction: International consensus statement	Cardio-respiratory	
Xue, Z. Sun, J. Li, T. Huang, Z. Chen, W.	2019	How to evaluate the clinical outcome of joint-preserving treatment for osteonecrosis of the femoral head: development of a core outcome set	Neurology, Orthopaedics & Trauma	
Audigé L., Schwyzer H.-K., SA CES Consensus Panel, Durchholz H.	2019	Core set of unfavorable events of shoulder arthroplasty: an international Delphi consensus process	Neurology, Orthopaedics & Trauma	
Joachim, Kariym C Farid-Kapadia, Mufiza Butcher, Nancy J Chee-a-tow, Alyssandra Monsour, Andrea Cohen, Eyal Mahant, Sanjay Guttmann, Astrid Offringa, Martin the Complex Care COS Consensus Meeting Group	2019	Core outcome set for children with neurological impairment and tube feeding	Gastroenterology	
Helen Ingoe - York Trials Unit/The James Cook University Hospital - Principal Investigator	2019	An international multi-stakeholder delphi consensus exercise to develop a core outcomes set (COS) for surgical fixation of rib fractures	Neurology, Orthopaedics & Trauma	

Will Eardley - The James Cook University Hospital/York Trials Unit - Clinical Supervisor<br>Amar Rangan<br>Catherine Hewitt - York Trials Unit - Supervisor<br>Catriona McDaid - York Trials Unit - Supervisor				
van Tol RR, Kimman ML, Melenhorst J, Stassen LPS, Dirksen CD, Breukink SO; Members of the steering group are co-authors of this study and can be found under the heading Collaborators.	2019	European Society of Coloproctology (ESCP) Core Outcome Set (COS) for haemorrhoidal disease: An international Delphi Study among healthcare professionals.	Gastroenterology	
Blackwood, B. et al	2019	A Core Outcome Set for Critical Care Ventilation Trials	Cardio-respiratory	
Kapil Sahnan1,2, Phil J Tozer1,2, Samuel O Adegbola1,2, Matthew J Lee3, Nick Heywood4, Angus G K McNair5, Daniel Hind6, Nuha Yassin1, Alan J Lobo3, Steven R Brown3, Shaji Sebastian7, Robin K S Phillips1,2, Phillip F C Lung1,2, Omar D Faiz1,2, Kay Crook1, Sue Blackwell8, Azmina Verjee8, Ailsa L Hart1,2, Nicola S Fearnhead9 the ENiGMA collaborators	2018	Developing a core outcome set for fistulising perianal Crohn's disease	Gastroenterology	
Agha, Riaz A. M.B.B.S., M.Sc.(Oxf.); Pidgeon, Thomas E. M.B.Ch.B.; Borrelli, Mimi R. M.B.B.S.; Dowlut, Naeem B.Sc.(Hons.); Orkar, Ter-Er K. M.B.Ch.B.; Ahmed, Maziyah B.Sc.; Pujji, Ojas; Orgill, Dennis P. M.D., Ph.D.; for the VOGUE Group	2018	Validated Outcomes in the Grafting of Autologous Fat to the Breast: The VOGUE Study. Development of a Core Outcome Set for Research and Audit	Cancer	
Benjamin Saul Raywood Allin<br>Nigel J Hall<br>Andrew R Ross	2018	Development of a gastroschisis core outcome set	Gastroenterology	

Sean S Marven<br>Jennifer J Kurinczuk<br>Marian Knight<br>on behalf of the NETS1G collaboration				
Singh, J. A. Dowsey, M. M. Dohm, M. Goodman, S. M. Leong, A. L. Scholte Voshaar, Mmjh Choong, P. F.	2017	Achieving Consensus on Total Joint Replacement Trial Outcome Reporting Using the OMERACT Filter: Endorsement of the Final Core Domain Set for Total Hip and Total Knee Replacement Trials for Endstage Arthritis	Neurology, Orthopaedics & Trauma	
Jasvinder A. Singh, Michael Dohm and Peter F. Choong	2017	Consensus on draft OMERACT core domains for clinical trials of Total Joint Replacement outcome by orthopaedic surgeons: a report from the International consensus on outcome measures in TJR trials (I-COMiTT) group	Neurology, Orthopaedics & Trauma	
PI – Benjamin Allin, NIHR funded Doctoral Research Fellow, National Perinatal Epidemiology Unit, and Paediatric Surgical Registrar, John Radcliffe Hospital, Oxford.<br><br>Marian Knight, NIHR Professor of Maternal and Child Population Health, Honorary Consultant in Public Health, National Perinatal Epidemiology Unit, Nuffield Department of Population Health	2017	NETS1HD study: development of a Hirschsprung's disease core outcome set	Gastroenterology	
Carina Benstoem ,<br>Ajay Moza,<br>Patrick Meybohm,<br>Christian Stoppe,<br>Rüdiger Autschbach,<br>Declan Devane,<br>Andreas Goetzenich	2017	A core outcome set for adult cardiac surgery trials: A consensus study	Cardio-respiratory	
Kerry N. L. Avery, PhD, Katy A. Chalmers, PhD, Sara T. Brookes, PhD, Natalie S. Blencowe, PhD,	2017	Development of a Core Outcome Set for Clinical Effectiveness Trials in Esophageal Cancer Resection Surgery	Cancer	

Karen Coulman, PhD, Katie Whale, DHealthPsy, Chris Metcalfe, PhD, y and Jane M. Blazeby, MD z, on behalf of the ROMIO Study Group, the CONSENSUS Esophageal Cancer Working Group				
PI: Marinus de Kleuver (Orthopaedic Surgery, VU Medical Center Amsterdam and St. Maartenskliniek Nijmegen)<br>CO-PI: David W. Polly Jr. (Orthopaedic Surgery, University of Minnesota)<br><br>Tsjitske Haanstra (Orthopaedic Surgery, VU Medical Center Amsterdam)<br>Miranda van Hooff (Research Department, St. Maartenskliniek Nijmegen)<br>Sayf Faraj (Orthopaedic Surgery, VU Medical Center Amsterdam)	2017	Defining a core outcome set for adolescent and young adult patients with a spinal deformity	Neurology, Orthopaedics & Trauma	
Stoner, M. C. Calligaro, K. D. Chaer, R. A. Dietzek, A. M. Farber, A. Guzman, R. J. Hamdan, A. D. Landry, G. J. Yamaguchi, D. J. Society for Vascular, Surgery	2016	Reporting standards of the Society for Vascular Surgery for endovascular treatment of chronic lower extremity peripheral artery disease	Cardio-respiratory	
Karen D. Coulman ,<br>James Hopkins,<br>Sara T. Brookes,<br>Katy Chalmers,<br>Barry Main,<br>Amanda Owen-Smith,<br>Robert C. Andrews,<br>James Byrne,	2016	A Core Outcome Set for the Benefits and Adverse Events of Bariatric and Metabolic Surgery: The BARIACT Project	Other	

Jenny L. Donovan,<br>Graziella Mazza,<br>Barnaby C. Reeves,<br>Chris A. Rogers,<br>Janice L. Thompson,<br>Richard Welbourn,<br>Sarah Wordsworth,<br>Jane M. Blazeby,<br>BARIACT working group				
Laurent Audigé<br>Matthias Flury<br>Andreas M. Müller<br>Holger Durchholz	2016	Complications associated with arthroscopic rotator cuff tear repair: definition of a core event set by Delphi consensus process	Neurology, Orthopaedics & Trauma	
Mr Andrew Currie- St Mark's Hospital, London<br>Prof Robin Kennedy- St Mark's Hospital, London, UK<br>Mr Omar Faiz - St Mark's Hospital, London, UK<br>Prof Ronan Cahill - University College Dublin, Ireland<br>Prof Conor Delaney - CASE Western Reserve University, Cleveland, USA<br>Dr Morris Franklin - Texas Endosurgery Institute, USA	2016	International expert consensus on endpoints for full-thickness laparoendoscopic colonic excision	Cancer	
van den Bos, W. Muller, B. G. de Bruin, D. M. de Castro Abreu, A. L. Chaussy, C. Coleman, J. A. Finelli, A. Gill, I. S. Gross, M. E. Jenniskens, S. F. Kahmann, F. Laguna-Pes, M. P. Rastinehad, A. R. Simmons, L. A. Sulser, T. Villers, A. Ward, J. F. de la Rosette, J. J.	2015	Salvage ablative therapy in prostate cancer: international multidisciplinary consensus on trial design	Cancer	

S. Potter1, C. Holcombe3, J. A. Ward1 and J. M. Blazeby1,2, on behalf of the BRAVO Steering Group*	2015	Development of a core outcome set for research and audit studies in reconstructive breast surgery	Cancer	
Imran Mahmud, MD, MPH; Thomas Kelley, MD, MBA; Caleb Stowell, MD; Aravind Haripriya, MD, MS; Anders Boman, MD; Ingrid Kossler, MBA; Nigel Morlet, FRANZCO, FRACS; Suzann Pershing, MD, MS; Konrad Pesudovs, PhD; Pik Pin Goh, MD, MS; John M. Sparrow, DPhil, FRCOphth; Mats Lundström, MD, PhD	2015	A Proposed Minimum Standard Set of Outcome Measures for Cataract Surgery	Ophthalmology	
van den Bos, W. Muller, B. G. Ahmed, H. Bangma, C. H. Barret, E. Crouzet, S. Eggener, S. E. Gill, I. S. Joniau, S. Kovacs, G. Pahernik, S. de la Rosette, J. J. Rouviere, O. Salomon, G. Ward, J. F. Scardino, P. T.	2014	Focal therapy in prostate cancer: international multidisciplinary consensus on trial design	Cancer	
Chiu, Alexander K. C. Din, Nizar Ali, Nadeem	2014	Standardising reported outcomes of surgery for intermittent exotropia--a systematic literature review	Ophthalmology	
Haywood, K. L. Griffin, X. L. Achten, J. Costa, M. L.	2014	Developing a core outcome set for hip fracture trials.	Neurology, Orthopaedics & Trauma	
Martin, N. E. Massey, L. Stowell, C. Bangma, C. Briganti, A. Bill-Axelson, A. Blute, M. Catto, J. Chen, R. C. D'Amico, A. V. Feick, G. Fitzpatrick, J. M. Frank, S. J. Froehner, M. Frydenberg, M. Glaser, A. Graefen, M. Hamstra, D. Kibel, A. Mendenhall, N. Moretti, K. Ramon, J. Roos, I. Sandler, H.	2014	Defining a Standard Set of Patient-centered Outcomes for Men with Localized Prostate Cancer	Cancer	

Sullivan, F. J. Swanson, D. Tewari, A. Vickers, A. Wiegel, T. Huland, H.				
Lynch, A. D. Logerstedt, D. S. Grindem, H. Eitzen, I. Hicks, G. E. Axe, M. J. Engebretsen, L. Risberg, M. A. Snyder-Mackler, L.	2013	Consensus criteria for defining 'successful outcome' after ACL injury and reconstruction: A Delaware-Oslo ACL cohort investigation	Neurology, Orthopaedics & Trauma	
Chen, R. C. Chang, P. Vetter, R. J. Lukka, H. Stokes, W. A. Sanda, M. G. Watkins-Bruner, D. Reeve, B. B. Sandler, H. M.	2014	Recommended patient-reported core set of symptoms to measure in prostate cancer treatment trials	Cancer	
Chera, B. S. Eisbruch, A. Murphy, B. A. Ridge, J. A. Gavin, P. Reeve, B. B. Bruner, D. W. Movsas, B.	2014	Recommended patient-reported core set of symptoms to measure in head and neck cancer treatment trials	Cancer	
Donovan, K. A. Donovan, H. S. Cella, D. Gaines, M. E. Penson, R. T. Plaxe, S. C. von Gruenigen, V. E. Bruner, D. W. Reeve, B. B. Wenzel, L.	2014	Recommended patient-reported core set of symptoms and quality-of-life domains to measure in ovarian cancer treatment trials	Cancer	
Reeve, B. B. Mitchell, S. A. Dueck, A. C. Basch, E. Cella, D. Reilly, C. M. Minasian, L. M. Denicoff, A. M. O'Mara, A. M. Fisch, M. J. Chauhan, C. Aaronson, N. K. Coens, C. Bruner, D. W.	2014	Recommended patient-reported core set of symptoms to measure in adult cancer treatment trials	Cancer	
Timaran, Carlos H. McKinsey, James F. Schneider, Peter A. Littooy, Fred	2011	Reporting standards for carotid interventions from the Society for Vascular Surgery	Cardio-respiratory	
Schumacher, H. Christian Meyers, Philip M. Higashida, Randall T. Derdeyn, Colin P. Lavine, Sean D. Nesbit, Gary M. Sacks, David Rasmussen, Peter Wechsler, Lawrence R.	2010	Reporting standards for angioplasty and stent-assisted angioplasty for intracranial atherosclerosis	Neurology, Orthopaedics & Trauma	
Conte, Michael S. Geraghty, Patrick J. Bradbury, Andrew W. Hevelone, Nathanael D. Lipsitz,	2009	Suggested objective performance goals and clinical trial design for evaluating catheter-based treatment of critical limb ischemia	Cardio-respiratory	

Stuart R. Moneta, Gregory L. Nehler, Mark R. Powell, Richard J. Sidawy, Anton N.				
Cross, Hugh	2005	A Delphi consensus on criteria for contraindications, assessment indicators and expected outcomes related to tibialis posterior transfer surgery	Other	
Nystrom, P. O., R. Bax, et al.	1990	Proposed definitions for diagnosis, severity scoring, stratification, and outcome for trials on intraabdominal infection.	Gastroenterology	
Nahmias, J. , Byerly, S. , Stein, D. , Haut, E. , Smith, J. , Gelbard, R. , Ziesmann, M. , Boltz, M. , Zarzaur, B. , Biffl, W. , Brenner, M. , DuBose, J. , Fox, C. , Galante, J. , Martin, M. , Moore, E. , Moore, L. , Morrison, J. , Norii, T. , Scalea, T. & Yeh, D.	2022	A core outcome set for resuscitative endovascular balloon occlusion of the aorta: A consensus based approach using a modified Delphi method	Neurology, Orthopaedics & Trauma	
Saskya Byerly, Jeffry Nahmias, Deborah M Stein, Elliott R Haut, Jason W Smith, Rondi Gelbard, Markus Ziesmann, Melissa Boltz, Ben L Zarzaur, Miklosh Bala, Andrew Bernard, Scott Brakenridge, Karim Brohi, Bryan Collier, Clay Cothren Burlew, Michael Cripps, Bruce Crookes, Jose J Diaz, Juan Duchesne, John A Harvin, Kenji Inaba, Rao Ivatury, Kevin Kasten, Jeffrey D. Kerby, Margaret Lauerman, Tyler Loftus, Preston R. Miller, Thomas Scalea, D Dante Yeh	2022	A core outcome set for damage control laparotomy via modified Delphi method	Neurology, Orthopaedics & Trauma	
Hajar Almoajil,Sally Hopewell,Helen Dawes,Francine Toye,Tim Theologis	2022	A core outcome set for lower limb orthopaedic surgery for children with cerebral palsy: An international multi-stakeholder consensus study	Neurology, Orthopaedics & Trauma	

Roulla Katiri, Deborah A. Hall, Derek J. Hoare, Kathryn Fackrell, Adele Horobin, Nicholas Hogan, Nóra Buggy, Paul H. Van de Heyning, Jill B. Firszt, Iain A. Bruce & Pádraig T. Kitterick for the Core Rehabilitation Outcome Set for Single-Sided Deafness (CROSSSD) initiative	2022	The Core Rehabilitation Outcome Set for Single-Sided Deafness (CROSSSD) study: International consensus on outcome measures for trials of interventions for adults with single-sided deafness	ENT	
Audigé, L.	2021	Core set of unfavorable events of proximal humerus fracture treatment defined by an international Delphi consensus process	Neurology, Orthopaedics & Trauma	
	2020	A core outcome set for future endometriosis research: an international consensus development study	Obstetrics and Gynecology	
Reynolds, K. A.	2019	Core Outcome Set for Actinic Keratosis Clinical Trials	Other	
Butler, D. P.	2020	An International Collaborative Standardizing Patient-Centered Outcome Measures in Pediatric Facial Palsy	Neurology, Orthopaedics & Trauma	
