# Supplementary material for: Continuity of outcome assessment throughout the lifecycle of surgical research: mapping core outcome domains measured in early phase and late phase studies
Source: BMC Surg. 2025 Oct 10;25:473. doi: 10.1186/s12893-025-03209-9 (PMC12512511; doi:10.1186/s12893-025-03209-9)
Supplement: Supplementary file 1 — Supplementary material 1. [file 12893_2025_3209_MOESM1_ESM.zip › Additional file 1 - COHESIVE COS.pdf]

Additional file 1

The COHESIVE Core outcome set

A recent multi-stakeholder, multi-national consensus study has agreed on a Core Outcome Set for surgical innovation to include eight core outcome domains for use in all early phase studies of new procedures and devices (the COHESIVE study). Routine use of the COHESIVE COS will enable efficient evaluation of surgical innovation throughout their translational pipeline until they become established into clinical practice. Within the context of surgical innovation, some core domains are particularly relevant (e.g. procedure success, modifications to the technique, operators' physical, emotional and psychological experiences). Other domains are relevant to both early and late phase (effectiveness) studies (e.g. anticipated advantages and disadvantages of the procedure/device).

Hoffmann C, Sewart E, Dodd S, Gorst SL, Blazeby JM, Avery KNL, Potter S, Macefield RC. Continuity of outcome assessment throughout the lifecycle of surgical research: mapping core outcome domains measured in early phase and effectiveness studies, *BMC Surgery.* Correspondence to: c.hoffmann@bristol.ac.uk (Bristol Medical School: Population Health Sciences, University of Bristol)

Core outcome set for surgical innovation

Modifications

Includes modifications to the
- (i) procedure,
- (ii) concomitant interventions or
- (iii) which patients were offered the procedure during the study

NB: excludes abandoning or changing to another procedure at any point (eg, laparoscopic approach converted to open)

Intended benefits

Refers to any intended benefit(s) of the procedure that occurred
- (i) before,
- (ii) during or
- (iii) after

the procedure (eg, fewer tests needed before surgery, less operative time, better recovery)

Procedure completion success

Describes the procedure completion success, either with or without modifications
(eg, planned innovative procedure was abandoned or changed, all technical steps were completed as planned)

Expected & unexpected disadvantages

Includes any of the following:

expected disadvantages that occurred
- (i) before,
- (ii) during or
- (iii) after

the procedure (eg, more tests needed before surgery, longer operative time, more patients required intensive care)

unexpected disadvantages that occurred
- (i) before,
- (ii) during or
- (iii) after

the procedure (eg, unexpected instrument clashing, inadvertent injury to nearby tissue and/or organs)

Problems with device working

Whether any problem(s) with the device working occurred, if applicable (eg, new stapler misfired)

Overall desired effect achieved

Whether the overall desired effect (overall aim) of the procedure/device was achieved (eg, tumour successfully excised)

Surgeons' experience

Surgeons'/operators' emotional, psychological, or physical experience of the procedure (eg, ergonomic comfort during the operation)

Patients' experience

Patients' emotional, psychological, or physical experience relating to the procedure being innovative (eg, anxiety because of the procedure being new)

Publication available at: Avery K. et al. (2021). A core Outcome Set for Seamless, Standardized Evaluation of Innovative Surgical Procedures and Devices (COHESIVE): A Patient and Professional Stakeholder consensus Study. *Annals of surgery*, 10.1097/SLA.0000000000004975.

cohesive
Core Outcomes for early pHasE Surgical Innovation and deVicEs study

University of BRISTOL

NIHR | Bristol Biomedical Research Centre
