# Supplementary material for: Continuity of outcome assessment throughout the lifecycle of surgical research: mapping core outcome domains measured in early phase and late phase studies
Source: BMC Surg. 2025 Oct 10;25:473. doi: 10.1186/s12893-025-03209-9 (PMC12512511; doi:10.1186/s12893-025-03209-9)
Supplement: Supplementary file 1 — Supplementary material 1. [file 12893_2025_3209_MOESM1_ESM.zip › Additional file 2 - supplemental information.pdf]

Additional file 2

Data extraction – supplemental information

This was piloted and refined using three studies prior to the full data extraction process. Extracted data included: i) descriptive study characteristics (e.g. author, publication year, journal), ii) clinical areas for which the COS is considered (e.g. type of invasive/surgical intervention, disease), iii) COS characteristics (e.g. COS description, target population, setting for intended use), vi) method of COS development (including methods for item generation and consensus) and v) verbatim core outcomes/domains included in the final COS. Information about outcome measurement was not extracted.

Where a study reported higher-level core outcome domains and lower-level core outcomes, data at the most granular level available was extracted (e.g. where 'pain' was the domain and 'abdominal pain' was included as particular type of pain, we extracted the lower-level outcome 'abdominal pain').

Table S1. Data extraction form

1	**Author**	
2	**Year**	
3	**Journal**	
4	**DOI**	
5	**COS description (verbatim)**	
6	**Study type (verbatim)**	COS for clinical trials or clinical research
		COS for registry
		COS for practice
		Recommendations for outcome measures
		Systematic review of outcomes measured in trials
		Definition
		COS patient reported outcomes
7	**Disease/ condition**	[obtained from COSMIN database]
8	**Intervention category**	Surgery
		Non-surgical interventional procedure
		Device
		Other invasive procedure
9	**Population**	Adults
		Children

Hoffmann C, Sewart E, Dodd S, Gorst SL, Blazeby JM, Avery KNL, Potter S, Macefield RC. Continuity of outcome assessment throughout the lifecycle of surgical research: mapping core outcome domains measured in early phase and effectiveness studies, *BMC Surgery.* Correspondence to: c.hoffmann@bristol.ac.uk (Bristol Medical School: Population Health Sciences, University of Bristol)

		Both
		Not specified
10	**Methodology for COS development (select all that apply)**<br>Select: 1 = for item generation; 2 = in consensus process; 3 = both	Literature review
		Systematic review
		Qualitative work
		Delphi
		Consensus meeting
		Survey
		Expert opinion
		Other
		If other, specify
11	**Patient involvement**<br>Select: 0 = No PPIE; 1 = for item generation; 2 = in consensus process; 3 = both	
12	**Number of items in COS** (including non-outcomes)	
13	**Core outcomes (verbatim)**	
14	**COHESIVE categorisation (definite match, possible match, no match** – *not mutually exclusive***)**	1 Benefits
		2 Modifications
		3 Completion success
		4 Problems with device
		5 Disadvantages
		6 Desired effect
		7 Surgeon experience
		8 Patient experience (relating to innovative nature)
15	**Overall grading**	Definite match to at least 1 COHESIVE domain
		Possible match to at least 1 COHESIVE domain
		No match
16	**Data reviewer's notes**	

Results – supplemental information

Table S2. Details about methods for item generation and consensus (n=54)

	n (%)
**Item generation only**	
Literature review	19 (35)
Systematic review	27 (50)
Qualitative work	12 (22)
Delphi	11 (20)
Consensus meeting	1 (2)
Survey	5 (9)
Expert opinion	14 (26)
Other	5 (9)
**Consensus process only**	
Delphi	19 (35)
Consensus meeting	21 (39)
Survey	4 (7)
Expert opinion	6 (11)
Other	2 (4)
**Item generation and consensus process**	
Qualitative work	1 (2)
Delphi	7 (13)
Consensus meeting	1 (2)
Expert opinion	4 (7)
Other	1 (2)
**Level of patient involvement in item generation or consensus**	
No patient involvement	20 (37)
Item generation only	2 (4)
Consensus process only	11 (20)
Item generation and consensus process	21 (39)

Table S3. Example later phase outcomes/domains categorised as “definite” or “possible match” to early phase core domains

COHESIVE core domain	‘Definite match’ verbatim extracted outcomes/domains	‘Possible match’ verbatim extracted outcomes/domains
**Intended benefits**	Able to walk normally (short distance)<br>Improvement in the most troublesome symptom<br>Maintain walking speed<br>Long-term survival 6 months after discharge<br>Active range of motion/Knee in flexion<br>Return to sports<br>Ability to function and complete daily tasks, perform work and leisure related activities<br>Self-image<br>Life participation<br>Swallowing<br>Stop using orthosis, brace, splint	Major adverse cardiovascular event<br>Cardiovascular disease<br>Mortality<br>Evidence of arthritic changes<br>Overall perioperative complications<br>Urinary retention<br>Alignment and/or occlusion<br>Pain due to abnormal sensation or non-painful stimulus<br>Major bleeding<br>Long-term patency (10-year)<br>Aspiration pneumonia
**Expected and unexpected disadvantages**	Reduced quality of life<br>Pain/discomfort<br>Anxiety/depression<br>Neonatal mortality<br>Impact on social situations<br>24-hour mortality<br>Ruptured aorta<br>Bleeding<br>Clinical failure<br>Adverse effects on teeth or tooth-supporting structures	Lung function<br>Return to work<br>Child physical health state<br>Sensation of bladder fullness<br>Global quality of life<br>Sport participation<br>Hip mobility
**Overall desired effect achieved**	Complete clearance of Actinic Keratosis<br>Persistent or recurrent intraabdominal infection<br>Treatment success<br>The presence/absence of a cholesteatoma in the first 5 years after surgical removal of cholesteatoma	long-term faecal incontinence<br>Live birth<br>Gait pattern<br>Range of movement of hip flexion

	Negative appendicectomy<br>Cardiac arrest after ED arrival	Revision surgery<br>PSA level
**Procedure completion success**	Conversion to other procedure<br>Outcome measures procedural - technical success<br>Inoperability	Secure closure of the excision site<br>Successful intubation rate<br>Need for adjunctive procedures
**Problems with the device working**	Device related complications<br>Tube malfunction/reoperation<br>Device events: events affecting any component of the implanted device or material or the instrumentation used for their implantation<br>Breakages<br>Technical complications of the specific operation	Device (Intra and post-operative)<br>Revision surgery<br>Revision<br>Reintubation or Failed extubation<br>Technique survival
**Operators'/surgeons' experience**		Ease of intubation<br>Effective communication between healthcare team and patient/carers
**Patients' experience**	Effective communication between healthcare team and patient/carers<br>Patient perspective on effectiveness<br>Patient-reported future treatment preference<br>Patient satisfaction with treatment	Patient Satisfaction (satisfaction with the outcome, satisfaction with the procedure)<br>Patient satisfaction<br>Impact on quality of life, such as self-esteem (feeling self-confident), emotional well-being (feelings of emotional and psychological health after surgery), normality (feeling 'back to normal self' or 'whole' as a result of surgery<br>Patient satisfaction<br>Patient-related adherence

Table S4. Later phase outcomes/domains categorised as “no match”

Verbatim extracted outcomes/domains
Social support
Social media/pictures
Education/support
Time to pregnancy leading to live birth
Gestational age at delivery
Birthweight
Days to fascial closure
Time to definitive hemorrhage control
Door-to-balloon inflation time
Time to achieve hemodynamic stability
Time to death
time from onset of treatment to the development of 3 organ failures
time to recovery
Suitability of Footwear/orthosis
Description of revascularization procedure
Preprocedure stenosis grade and length
Postprocedure stenosis grade and length
Pharmacologic adjuncts
Hospital days
Intensive care unit days
Postprocedural stratifcation by symptoms, high-risk categories
Annualized rate of stroke
Assessment of cost of new technology
Competition of interest and conflict of interest
CAS techniques and procedures
type of anesthesia
description of the surgical technique
all conditions presumed to affect reported outcomes
Treatment approaches included
Baseline characteristics
Pain description
number and location of cores taken
number of positive cores
amount per biopsy of the involved cancer (in millimetres)
selection failure
presence of malignancy in the specimen
micronutrient status
Patient Participation
Number of operations
Number of fat grafting sessions needed to get optimal result as judged by the patient and/or surgeon (who made the judgement should be stated
The incidence of radiological abnormalities expressed quantitatively (e.g. as a number and a percentage)

Any interference with subsequent mammography scanning expressed quantitatively (e.g. as a number and a percentage)
Duration of stay: time from randomisation until patient first leaves the relevant facility or dies
Ventilation
Invasive mechanical ventilation (resource use)
Days spent admitted to hospital/intensive care per year
Frequency of doctor/clinic/emergency room visits per year (as related to frequency of illness)
Cause of death
Effectiveness of pain relief
Length of hospital stay
Acetabular coverage and hip congruency
Femoral head shape
Hospital length of stay
Exact specifications of the patient group
Exact specifications of the device used
Hospital length of stay
Exact specifications of the patient group
Exact specifications of the device used
