# Supplementary material for: Continuity of outcome assessment throughout the lifecycle of surgical research: mapping core outcome domains measured in early phase and late phase studies
Source: BMC Surg. 2025 Oct 10;25:473. doi: 10.1186/s12893-025-03209-9 (PMC12512511; doi:10.1186/s12893-025-03209-9)
Supplement: Supplementary file 1 — Supplementary material 1. [file 12893_2025_3209_MOESM1_ESM.zip › Additional file 3 - Mapping manual.pdf]

Additional file 3

Instructions for mapping outcomes/domains

Mapping

Outcomes should be mapped to as many domains as may be relevant, which may include several. Each mapping is rated either definite match or possible match.

- Outcomes that clearly relate to a COHESIVE domain should be mapped as 'definite'.
- Items may be mapped as 'possible match' if the mapping may depend on the interpretation of the outcome meaning or may be context-dependent.
- The choice between the two grades of mapping requires a degree of judgement by the reviewer.

Each outcome receives an overall grade of mapping, using a traffic light system:

- Green = 'definite match' to at least one domain
- Amber = 'possible match' to at least one domain. No definite matches.
- Red = no match to any domain.

Positive vs. negative outcomes

Unless the authors specify a direction, the reviewer should make a judgement on whether the outcome is positive or negative.

- If felt to be a positive outcome, it should be a definite match to 'benefit' and possible match to 'disadvantage'
- If felt to be a negative outcome, it should be a definite match to 'disadvantage' and possible match to 'benefit'

If the authors specify a direction, then the mapping should simply be a definite match to the appropriate domain.
e.g. 'Death' = definite match to disadvantage; possible match to benefit
e.g. 'Reduced mortality rate' = definite match to benefit (since direction is specified)

Benefit vs. desired effect

Desired effect refers to the overall immediate aim of a technique or procedure e.g. removing a tumour, replacing someone's renal function, securing an airway.
Long term benefits e.g. reduced mortality are more relevant for domain one (benefit).

Hoffmann C, Sewart E, Dodd S, Gorst SL, Blazeby JM, Avery KNL, Potter S, Macefield RC. Continuity of outcome assessment throughout the lifecycle of surgical research: mapping core outcome domains measured in early phase and effectiveness studies, *BMC Surgery.* Correspondence to: c.hoffmann@bristol.ac.uk (Bristol Medical School: Population Health Sciences, University of Bristol)

Exemplary template

Core outcomes (verbatim)	**COHESIVE mapping**<br>Please put "1" in all relevant columns (each outcome may map to more than one domain)								**Overall grade of mapping**<br>Please put "1" in one column only		
	1<br>Benefits	2<br>Modifications	3<br>Completion success	4<br>Problems with device	5<br>Disadvantages	6<br>Desired effect	7<br>Surgeon experience	8<br>Patient experience (relating to innovative nature)	Definite match to at least 1 domain	Possible match to at least 1 domain	No match
